# Brain network mechanism on cognitive control task in the elderly with brain aging: A functional near infrared spectroscopy study

**DOI:** 10.3389/fnhum.2023.1154798

**Published:** 2023-03-15

**Authors:** Junjie Liang, Jiali Huang, Zhiwei Luo, Yuxi Wu, Lingxiong Zheng, Zengquan Tang, Wanying Li, Haining Ou

**Affiliations:** ^1^Department of Rehabilitation, The Fifth Affiliated Hospital of Guangzhou Medical University, Guangzhou, China; ^2^Department of Rehabilitation, Guihang Group 302 Hospital, Anshun, China; ^3^The Fifth Clinical College, Guangzhou Medical University, Guangzhou, China; ^4^Key Laboratory of Biological Targeting Diagnosis, Therapy, and Rehabilitation of Guangdong Higher Education Institutes, Guangzhou, China

**Keywords:** brain aging, cognitive control, functional near-infrared spectroscopy, brain network, near-infrared spectroscopy

## Abstract

**Objective:**

To study the brain network mechanism of cognitive control in the elderly with brain aging.

**Materials and methods:**

21 normal young people and 20 elderly people were included in this study. Mini-mental State Examination and functional near-infrared spectroscopy (fNIRS) synchronous judgment test (including forward tests and reverse judgment tests) were performed on all subjects. To observe and compare differences in brain region activation and brain functional connectivity between subjects and forward and reverse trials by recording functional connectivity (FC) in different task paradigms and calculating bilateral prefrontal and primary motor cortical (PMC) areas.

**Results:**

In the forward and reverse judgment tests, the reaction time of the elderly group was significantly longer than the young group (*P* < 0.05), and there was no significant difference in the correct rate. In the homologous regions of interest (ROI) data, the FC of PMC and prefrontal cortex (PFC) in the elderly group was significantly decreased (*P* < 0.05). In the heterologous ROI data, except for left primary motor cortex (LPMC)-left prefrontal cortex (LPFC), the other PMC and PFC of the elderly group were significantly lower than the young group (*P* < 0.05) while processing the forward judgment test. However, the heterologous ROI data of LPMC-right prefrontal cortex (RPFC), LPMC-LPFC and RPFC-LPFC in the elderly group were significantly lower than the young group (*P* < 0.05) while processing the reverse judgment test.

**Conclusion:**

The results suggest that brain aging affected degeneration of whole brain function, which reduce the speed of information processing and form a brain network functional connection mode different from that of young people.

## 1. Research background of cognitive control and brain aging

Cognitive control refers to the fundamental ability to ensure goal-oriented behaviors, including task switching, reaction inhibition and working memory. It is a broader concept than selective attention (Parris et al., 2022). Cognitive control participates in many traditional cognitive dimensions, including attention, execution and memory (Harada et al., 2013). People’s behavior is highly adaptable and flexible, and even if environmental factors interfere, people can make adjustments within a certain range and finally achieve their goals. Behind this flexibility is the role of cognitive control (Schuch et al., 2022). Selective attention and response inhibition in cognitive control can be effectively monitored by cognitive psychological monitoring methods (Braem et al., 2019; Mäki-Marttunen et al., 2019).

The related manifestations of cognitive control decreased will appear in the early stage of senile brain aging (Bettio et al., 2017). The decline of cognitive function will gradually develop into cognitive impairment-related diseases, such as mild cognitive impairment, Alzheimer’s disease (AD), dementia, etc., which will lead to the decline of life quality, aggravate neuropsychiatric symptoms and disabilities, and increase medical costs (Zheng et al., 2018). Early monitoring and intervention of cognition can effectively delay the processing of dementia and AD (Levin et al., 2010). Therefore, it is helpful for early screening and treatment to study the brain mechanism of cognitive control dysfunction caused by early brain aging (Zammit et al., 2016). In this study, functional near-infrared spectroscopy (fNIRS) was used to monitor the dynamic changes of brain network in normal elderly people during judgment test, so as to explore the influence mechanism of age-induced brain degeneration on cognitive and motor areas of prefrontal cortex.

## 2. Experimental design

### 2.1. Research object

In this study, from January 2022 to April 2022, 20 normal elderly and 21 normal young people were recruited in the Fifth Affiliated Hospital of Guangzhou Medical University. Inclusion criteria for healthy elderly: ➀ age 50–75 years; ➁ no previous history of vital organ disease; ➂ no brain tumor or other brain disease; ➃ right-handed; ➄ normal intellectual and cognitive ability; ➅ no history of mental illness. Exclusion criteria: ➀ Severe cognitive impairment or mental illness, unable to cooperate with instructions or tests; ➁ skull defect, head infection and skin damage are impossible for near-infrared brain imaging. Inclusion criteria for healthy young adults: ➀ age 18–30 years; ➁ no previous history of vital organ disease; ➂ no brain tumor or other brain disease; ➃ right-handed; ➄ normal intellectual and cognitive ability; ➅ no history of mental illness. All subjects have signed the informed consent form for the experiment. The research scheme has been approved by the Administrative Committee of the Fifth Affiliated Hospital of Guangzhou Medical University (KY01-2021-03-20) and registered by China Clinical Trial Center (ChiCTR2200058088).

### 2.2. Experimental methods

#### 2.2.1. Clinical evaluation

In this study, all subjects were assessed by Mini-Mental State Examination (MMSE) and fNIRS synchronous judgment test.

#### 2.2.2. Experimental equipment and fNIRS scheme setting

**FIGURE 1:**
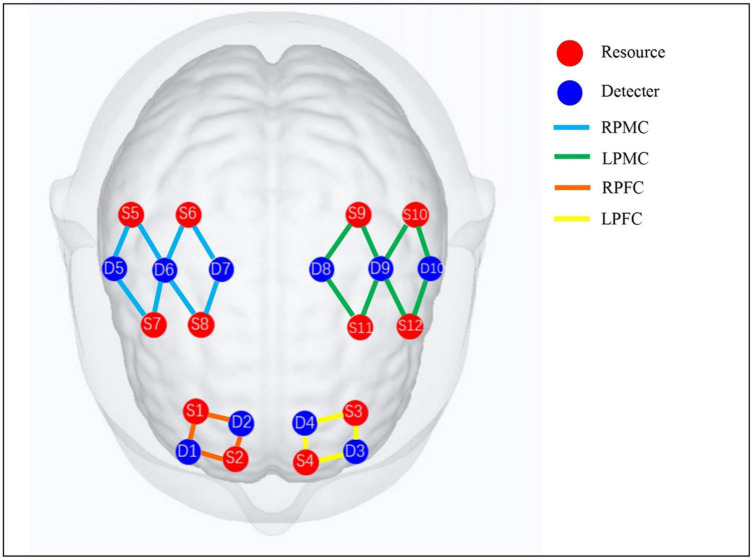
fNIRS observation channel distribution and ROI division diagram. RPMC, right primary motor cortex; LPMC, left primary motor cortex; RPFC, right prefrontal cortex; LPFC, left prefrontal cortex. PMC includes primary motor cortex, anterior motor cortex, and auxiliary motor cortex; PFC includes dorsolateral prefrontal cortex and frontal pole.

The NirSmart (Danyang Huichuang, China), a portable fNIRS imaging device of the Huichuang, was used to collect the data of the subjects during the task. Two kinds of near-infrared (730, 850 nm) signals with different wavelengths were recorded in the form of continuous wave, and the sampling frequency was 11 Hz. The fNIRS acquisition head cap consists of 10 detectors and 12 transmitters, with a total of 24 channels distributed in regions of interest (ROI). The detectors and transmitters placing followed by the International 10–20 system. The ROI is set in bilateral prefrontal cortex (PFC) and primary motor cortex (PMC), as shown in Figure 1. The channel distribution adopts the positioning system Nirspace (Danyang Huichuang Medical Equipment Co., Ltd., Danyang, China) of Polhemus FASTRAK 3D digital instrument to locate the brain area. After entering the evaluation room, the subjects sat comfortably for 5 min, got familiar with the environment, and eliminated the tension. The fNIRS synchronous judgment test scheme is designed as Block. Firstly, the resting state of 10 s is collected as the baseline. Then, the task state during the trial was collected for 60 s, and then the rest state data was collected for 60 s. As a trial, the trial was repeated for three times, totaling 370 s.

#### 2.2.3. Judgment of experimental design

**FIGURE 2:**
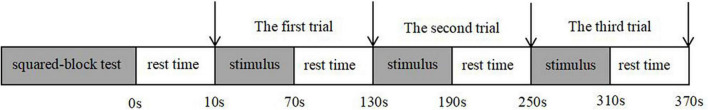
Flow chart of the judgment trial design.

The judgment task paradigm adopted in this study is squared-block test, including forward test and reverse test. Subjects need to click the “←” or “→” keyboard button according to the test content and the position of the font on the screen in the squared-block. When judging the forward test, the subject are required to click the key in the same position as the font in the squared-block; In the reverse judgment test, the subject are required to click the key in the opposite position as the font in the squared-block. The picture of squared-block test is presented for 4,000 ms, and then the cross gaze point is presented for 2,000 ms, this is a complete stimulus. A trial consists of 10 stimulus, and three trials are conducted continuously, every test interval has a rest time of 60 s. After completion, the trial ends (Figure 2). Squared-block test is a kind of research on cognitive psychology, mainly involves executive control, attention, language, cognition and other fields, usually around the internal mechanism of Stroop effect, or as a trial means, to explore a variety of psychological and cognitive problems (Yoo et al., 2020).

#### 2.2.4. fNIRS synchronous judgment test flow

After the researcher puts fNIRS on the subjects and determines the effectiveness of the channel, the subjects are instructed to adjust their sitting position. The forefinger and ring finger of the right hand are placed on the “←” and “→” keyboards respectively, and only clicking on the keyboard is required during the experiment. The experimental environment should be kept quiet all the time, and the limbs and head should not be moved at will.

At the beginning of the experiment, an instruction appeared, and the experimenter instructed the subjects to read the instruction and practice. The practice lasted for five complete stimuli, which ensured that the subjects understood the experimental process and could cooperate with the completion of the experiment. After the exercise, the formal test was started, and the forward and reverse judgment tests were carried out. fNIRS synchronously collected the hemoglobin changes of ROI through the marker. During this period, the key reaction time and accuracy rate of subjects were recorded. Carry out forward test first, and then reverse test.

### 2.3. fNIRS data processing

#### 2.3.1. Use NirSpark software to pre-process fNIRS data

➀ Remove of time interval and motion artifacts; ➁ Converting of the original optical signal into an optical density curve; ➂ Band-pass filtering (0.1–0.2 Hz) to filter the physiological noise caused by heart rate, respiration, exercise and the physiological noise caused by motion artifacts (Tan et al., 2015). Overall, in order to remove the low-frequency elements from the original signal, a sixth-order Butterworth band-pass filter was used to obtain the 0.1–0.2 Hz filter signal. Then, the sliding averaging method is used to remove the signal noise to improve the signal-to-noise ratio (Huppert et al., 2009); ➃ Calculate the concentration of oxygenated hemoglobin and deoxyhaemoglobin by the Hemo module; ➄ Calculate the eigenvalues of the forward and backward judgment tests with Block design (Yang et al., 2022).

#### 2.3.2. Functional connectivity (FC) analysis

Functional connectivity (FC) refers to the temporal correlation between distant neurophysiological events in space. Brain imaging technology can be used to detect the functional communication between multiple brain regions (Zheng et al., 2021). In the Network module of NirSpark software, the changes in HbO_2_ and HbR concentrations in subjects at each time point measured at rest were extracted, and the Pearson correlation coefficient of HbO_2_ and HbR contents in each channel in the time series was analyzed. Fisher Z-transformation was then performed, and the transformed value was defined as the FC strength between channels. FC data were processed by NirSpark software and corrected by false discovery rate (FDR).

Functional near-infrared spectroscopy (FNIRS) brain network data processing: ➀ Define ROI in the functional brain network analysis module as bilateral PFC area (4 channels left and right) and bilateral PMC area (8 channels left and right); ➁ Calculate the mean value of oxygenated hemoglobin signal in the ROI brain region; ➂ Calculate the functional connectivity (FC) data of homologous brain regions and heterologous brain regions.

### 2.4. Statistical analysis

SPSS 25.0 software was used for statistical analysis (Tak and Ye, 2014), and GraphPad 8.0 was used to draw data results. All subjects’ MMSE, FC value of brain network, reaction time of judgment test and correct rate data were tested normally, and all measurement data were expressed by mean standard deviation. The MMSE, the FC value of brain network, the correct rate of judgment test and the reaction time data between the two groups which accord with the normal distribution were tested by independent sample *t*-test, and comparison of data within the group were tested by paired *t*-test. For the data that does not conform to the normal distribution, Wilcoxon symbolic rank sum test should be used in intra-group statistics, and Mann-Whitney *U* test should be used in inter-group statistics. Statistical significance difference is defined as *P* < 0.05.

## 3. Results

### 3.1. General information

A total of 41 subjects were included in this study, including 21 young people with an average age of 21.476 ± 0.928 years; 20 elderly people, with an average age of 59.550 ± 8.401 years; The MMSE of the elderly group was significantly lower than that of the young group (*Z* = 3.389, *P* = 0.001), but all were in the normal range (see Table 1).

**TABLE 1:** Comparison of general data of two groups of subjects.

Group	*n*	Gender (male/female)	Age (years)	MMSE score
Young group	22	11/10	21.476 ± 0.928	29.238 ± 0.995
Elderly group	20	8/12	59.550 ± 8.401	26.900 ± 2.673
*t/Z* value			−5.514^#^	3.389^#^
*P*-value			<0.001	0.001

^#^Rank sum test is adopted.

### 3.2. Within-group and between-group differences in behavioral data for forward and reverse judgment tests

**FIGURE 3:**
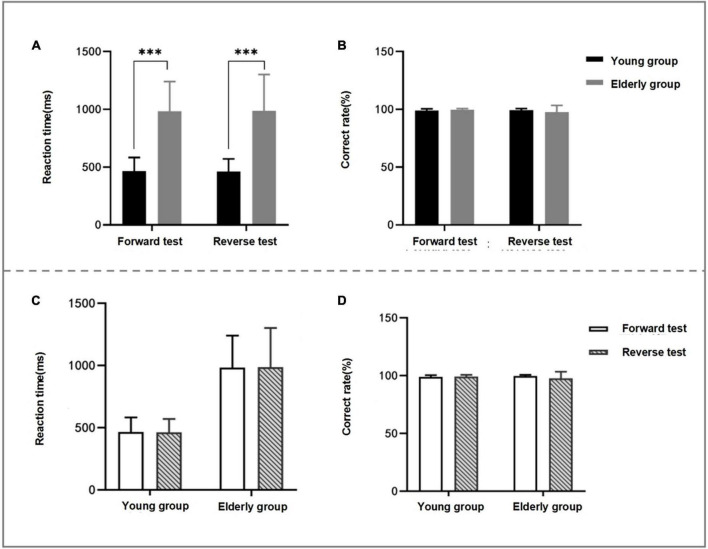
Comparison between groups and within groups of behavioral data of two groups of subjects’ judgment test. **(A,B)** Are the comparison between two groups of subjects’ judgment test behavior data, panel **(A)** is the comparison between groups at reaction time, and panel **(B)** is the comparison between groups at correct rate. **(C,D)** Shows the intra-group comparison of behavioral data of two groups of subjects’ judgment test, panel **(C)** is the intra-group comparison of reaction time and panel **(D)** is the intra-group comparison of correct rate; as can be seen from panel **(A)**, the reaction time of the elderly group is significantly longer than that of the young group in the forward and reverse judgment tests (*P* < 0.05). *P < 0.05; ^**^P < 0.01; ^***^P < 0.001.

In the forward and reverse judgment tests, the reaction time of the elderly group was significantly longer than that of the young group (forward *Z* value = −5.269, *P* < 0.001; reverse *Z* value = −5.112, *P* < 0.001). However, there was no statistical difference between the two groups in the correct rate of forward and reverse tests. There was no statistical difference in the reaction time and the correct rate within the two groups of subjects in the forward and reverse tests (see Table 2 and Figure 3).

**TABLE 2:** Comparison of reaction time and correct rate of judgment test between young and elderly.

	Group	Forward	Reverse	*t/Z* value	*P*-value
Reaction time (ms)	Young group	466.140 ± 116.478	462.616 ± 108.381	0.087^#^	0.931
	Elderly group	983.368 ± 257.843	987.428 ± 313.955	−0.120	0.906
	*t/Z* value	−5.269^#^	−5.112^#^		
	*P*-value	< 0.001	< 0.001		
Correct rate (%)	Young group	98.889 ± 1.610	99.206 ± 1.455	0.816^#^	0.414
	Elderly group	99.667 ± 1.026	97.639 ± 5.853	1.549^#^	0.121
	*t/Z* value	−1.782^#^	0.348^#^		
	*P*-value	0.075	0.728		

^#^Rank sum test is adopted, and the rest are *t*-test.

### 3.3. Differences between groups of brain networks in forward judgment test

**FIGURE 4:**
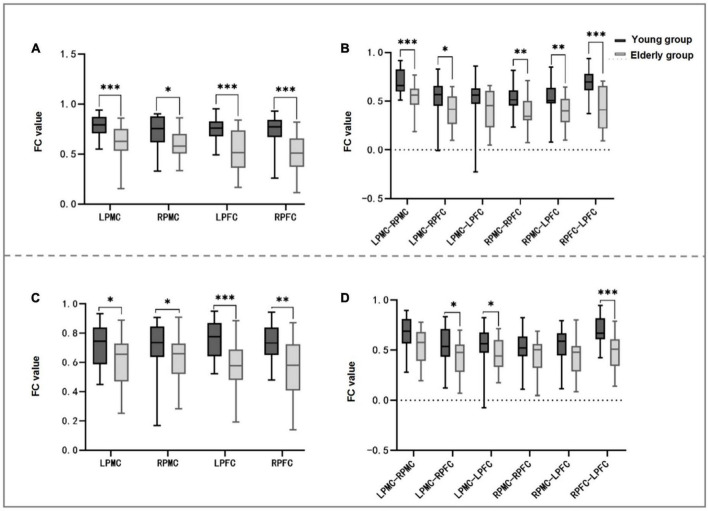
Comparison between groups of FC of homologous and heterologous ROI in forward and reverse judgment test. **(A,B)** Shows the forward judgment test, panel **(A)** is the inter-group comparison of FC values of homologous ROI between young and elderly, and panel **(B)** is the inter-group comparison of heterologous ROI. **(C,D)** Shows the comparison between groups of homologous ROI and heterologous ROI in the reverse judgment test. It can be seen from panels **(A,C)** that the FC values of homologous ROI between the two groups are statistically different in the forward and reverse judgment tests (*P* < 0.05). Panel **(B)** shows that the FC values of LPMC-RPMC, LPMC-RPFC, RPMC-RPFC, RPMC-LPFC, and RPFC-LPFC in the elderly group are significantly lower than those in the young group (*P* < 0.05). It can be seen from panel **(D)** that the FC values of LPMC-RPFC, LPMC-LPFC and RPFC-LPFC in the elderly group are significantly lower than those in the young group (*P* < 0.05). FC, functional connectivity; LPFC, left prefrontal cortex; RPFC, right prefrontal cortex; LPMC, left primary motor cortex; RPMC, right primary motor cortex; ROI, region of interest; *P < 0.05; ^**^P < 0.01; ^***^P < 0.001.

In the comparison of homologous brain networks between the two groups, the FC values of LPMC (*t* = 3.647, *P* = 0.001), RPMC (*Z* = 2.426, *P* = 0.015), LPFC (*t* = 4.255, *P* < 0.001), and RPFC (*Z* = 3.678, *P* < 0.001) in the elderly group were significantly lower than those of the young group (see Table 3 and Figure 4A).

**TABLE 3:** Comparison of functional connectivity (FC) values of homologous and heterologous brain networks between the two groups in forward judgment test.

	ROI	Young group	Elderly group	*t/Z* value	*P*-value
Homologous ROI	LPMC	0.779 ± 0.121	0.610 ± 0.173	3.647	0.001
	RPMC	0.720 ± 0.167	0.598 ± 0.140	2.426	0.015^#^
	LPFC	0.753 ± 0.111	0.527 ± 0.211	4.255	<0.001
	RPFC	0.737 ± 0.155	0.512 ± 0.188	3.678^#^	<0.001
Heterologous ROI	LPMC-RPMC	0.704 ± 0.128	0.547 ± 0.140	3.727	0.001
	LPMC-RPFC	0.547 ± 0.189	0.397 ± 0.171	2.668	0.011
	LPMC-LPFC	0.537 ± 0.218	0.425 ± 0.187	1.930	0.054^#^
	RPMC-RPFC	0.532 ± 0.163	0.384 ± 0.154	2.988	0.005
	RPMC-LPFC	0.539 ± 0.175	0.399 ± 0.151	2.737	0.009
	RPFC-LPFC	0.683 ± 0.136	0.431 ± 0.215	4.473	<0.001

^#^Rank sum test is adopted, and the rest are *t*-test.

In the comparison of heterologous brain network between the two groups, the FC values of LPMC-RPMC (*t* = 3.727, *P* = 0.001), LPMC-RPFC (*t* = 2.668, *P* = 0.011), RPMC-RPFC (*t* = 2.988, *P* = 0.005), RPMC-LPFC (*t* = 2.737, *P* = 0.009), RPFC-LPFC (*t* = 4.473, *P* < 0.001) in the elderly group were significantly lower than those of the young group, and there was no statistical difference in LPMC-LPFC between the two groups (see Table 3 and Figure 4B).

### 3.4. Differences between groups of brain networks in reverse judgment experiment

In the comparison of homologous brain networks between the two groups, the FC values of LPMC (*t* = 2.609, *P* = 0.013), RPMC (*Z* = 2.008, *P* = 0.045), LPFC (*t* = 3.792, *P* < 0.001), and RPFC (*Z* = 3.284, *P* < 0.003) in the elderly group were significantly lower than those of the young group (see Table 4 and Figure 4C).

**TABLE 4:** Comparison of functional connectivity (FC) values of homologous and heterologous brain networks between the two groups in reverse judgment test.

	ROI	Young group	Elderly group	*t/Z* value	*P*-value
Homologous ROI	LPMC	0.727 ± 0.142	0.600 ± 0.169	2.609	0.013
	RPMC	0.710 ± 0.182	0.626 ± 0.153	2.008	0.045^#^
	LPFC	0.755 ± 0.130	0.568 ± 0.181	3.792	<0.001
	RPFC	0.731 ± 0.132	0.548 ± 0.213	3.284	0.003
Heterologous ROI	LPMC-RPMC	0.658 ± 0.178	0.553 ± 0.167	1.956	0.058
	LPMC-RPFC	0.549 ± 0.167	0.423 ± 0.167	2.413	0.021
	LPMC-LPFC	0.548 ± 0.181	0.452 ± 0.160	2.113	0.035^#^
	RPMC-RPFC	0.512 ± 0.199	0.450 ± 0.167	1.085	0.285
	RPMC-LPFC	0.542 ± 0.174	0.437 ± 0.170	1.956	0.058
	RPFC-LPFC	0.688 ± 0.143	0.480 ± 0.169	4.260	<0.001

^#^Rank sum test is adopted, and the rest are *t*-test.

In the comparison of heterologous brain network between the two groups, the FC values of LPMC-RPFC (*t* = 2.413, *P* = 0.021), LPMC-LPFC (*Z* = 2.113, *P* = 0.035), and RPFC-LPFC (*t* = 4.260, *P* < 0.001) in the elderly group were significantly lower than those of the young group, and there was no statistical difference in LPMC-RPPMC, RPMC-RPFC, RPMC-LPFC between the two groups (see Table 4 and Figure 4D).

### 3.5. Intra-group differences of FC values of brain networks in forward and reverse judgment tests

**FIGURE 5:**
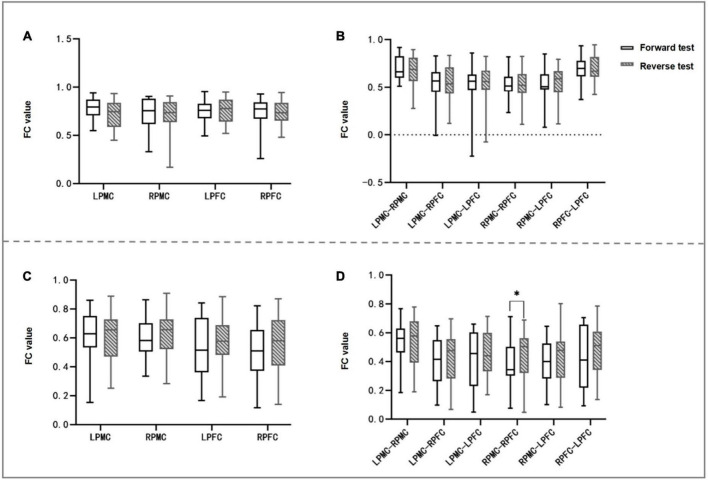
Intra-group comparison of FC of homologous and heterologous ROI of two groups of subjects. **(A,B)** Are young groups, panel **(A)** is the intra-group comparison of FC values of forward and reverse judgment tests of homologous ROI, and panel **(B)** is the intra-group comparison of heterologous ROI; **(C,D)** are the elderly group, panel **(C)** is the intra-group comparison of homologous ROI, and panel **(D)** is the intra-group comparison of heterologous ROI; panel **(D)** shows that the FC value of RPMC-RPFC is statistically different between the forward and reverse judgment test (*P* < 0.05). FC, functional connection; LPFC, left prefrontal cortex; RPFC, right prefrontal cortex; LPMC, left primary motor cortex; RPMC, right primary motor cortex; ROI, region of interest; *P < 0.05.

**FIGURE 6:**
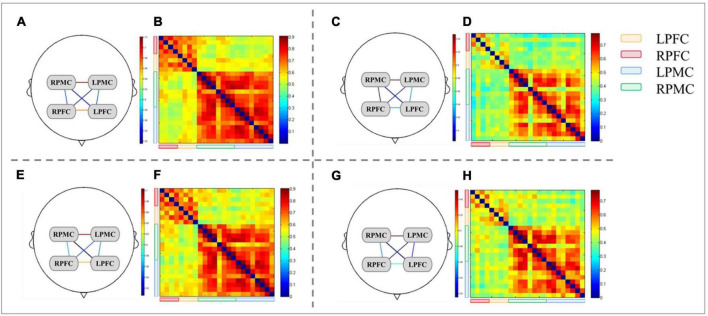
Brain network and FC matrix of two groups of subjects. **(A,B,E,F)** are young group, and **(C,D,G,H)** are elderly group; Panels **(A–D)** are forward judgment tests; Panels **(E–H)** are reverse judgment tests.

In the comparison between forward and reverse test brain networks, the FC value of the heterologous brain network RPMC-RPFC in the elderly group in the reverse test was significantly higher than that in the forward test (*t* value = −2.764, *P* = 0.012), and FC values of the other homologous ROI and heterologous ROI had no statistical difference within the group. There was no statistical difference in the FC value of brain network of young people in the forward and reverse judgment tests (see Table 5 and Figures 5, 6).

**TABLE 5:** Intra-group differences of functional connectivity (FC) values of brain network in forward and reverse judgment tests.

	ROI	Forward	Reverse	*t/Z* value	*P*-value
Young group homologous ROI	LPMC	0.779 ± 0.121	0.727 ± 0.142	1.899	0.072
	RPMC	0.720 ± 0.167	0.710 ± 0.182	0.608	0.543^#^
	LPFC	0.753 ± 0.111	0.755 ± 0.130	−0.950	0.925
	RPFC	0.737 ± 0.155	0.731 ± 0.132	0.226	0.821^#^
Young group heterologous ROI	LPMC-RPMC	0.704 ± 0.128	0.658 ± 0.178	1.93	0.068
	LPMC-RPFC	0.547 ± 0.189	0.549 ± 0.167	−0.084	0.934
	LPMC-LPFC	0.537 ± 0.218	0.548 ± 0.181	0.017	0.986^#^
	RPMC-RPFC	0.532 ± 0.163	0.512 ± 0.199	0.765	0.453
	RPMC-LPFC	0.539 ± 0.175	0.542 ± 0.174	−0.158	0.876
	RPFC-LPFC	0.683 ± 0.136	0.688 ± 0.143	−0.223	0.826
Elderly group homologous ROI	LPMC	0.610 ± 0.173	0.600 ± 0.169	0.519	0.610
	RPMC	0.598 ± 0.140	0.626 ± 0.153	−1.379	0.184
	LPFC	0.527 ± 0.211	0.568 ± 0.181	−1.323	0.201
	RPFC	0.512 ± 0.188	0.548 ± 0.213	−1.160	0.260
Elderly group heterologous ROI	LPMC-RPMC	0.547 ± 0.140	0.553 ± 0.167	−0.255	0.802
	LPMC-RPFC	0.397 ± 0.171	0.423 ± 0.167	−1.022	0.319
	LPMC-LPFC	0.425 ± 0.187	0.452 ± 0.160	−1.035	0.314
	RPMC-RPFC	0.384 ± 0.154	0.450 ± 0.167	−2.764	0.012
	RPMC-LPFC	0.399 ± 0.151	0.437 ± 0.170	−1.413	0.162
	RPFC-LPFC	0.431 ± 0.215	0.480 ± 0.169	−1.035	0.174

^#^Rank sum test is adopted, and the rest are *t*-test.

## 4. Discussion

Brain aging without neurodegenerative diseases, usually called non-pathological brain aging or normal cognitive aging, is characterized by impaired memory and cognitive function. With the increase of age, brain function gradually deteriorates, and age-related cognitive impairment accumulates, which increases the risk of neurodegenerative diseases (Borrás Blasco and Viña Ribes, 2016; Mattson and Arumugam, 2018). An important aspect of cognitive control is reaction inhibition, that is, information processing is performed correctly while interference information is suppressed. Reaction inhibition is affected by the gradual decline of cognitive ability in the process of aging (Tafuro et al., 2019). In this study, fNIRS synchronous judgment test was used to explore the mechanism of the influence of age on cognitive control behavior and brain network.

### 4.1. The influence of brain aging on judging test results

The forward judgment test only needs to perform the consistent key pressing task according to the picture presentation direction, which belongs to automatic processing, while the reverse judgment test needs to suppress the trend of processing in the same direction and then perform reverse processing. The results of this study show that the reaction time of the elderly group is significantly longer than that of the young group, but there is no statistical difference between the two groups in the correct rate. It should be noted that the MMSE scores of the elderly group are all at a normal level, suggesting that although the cognitive function is at a normal level, cognitive function has deteriorated in behavior, mainly in executive function, information processing, selective attention and reaction inhibition. Compared with young people, the reaction time of the old people’s judgment test is prolonged, and the consideration is caused by the slow speed of information processing. In the process of brain aging, the slowdown of information processing speed is a common manifestation. Yano (2011) conducted Simon test on the old and young people, the results showed that the reaction time of the old people was significantly longer than that of the young people. It was found that brain aging might reduce the speed of information processing, but it did not affect the inhibition of reaction, which was consistent with the behavioral results of this study. However, there is no obvious difference between the reaction time and the correct rate in the forward and reverse behavioral comparison of the old people’s judgment test. The reasons are as follows: ➀ The ability of automatic information processing is degraded. Information processing is mainly divided into automatic processing and controlled processing. Automatic processing does not require the participation of attention, while controlled processing needs extra attention (Li et al., 2022). The forward test belongs to automatic processing, while the reverse test belongs to controlled processing. As the elderly get older, their ability to process information automatically deteriorates, which may require longer reaction time to process information; ➁ Impairment of response inhibition function: manifested by the ability to inhibit irrelevant information decreases (Commodari and Guarnera, 2008; Nicosia and Balota, 2020); ➂ Setting the difficulty of judgment test: the difficulty and intensity of judgment test will affect cognitive control and cognitive reserve consumption; ➃ Attention deficit: Attention is the key ability to extract relevant information, and attention deficit can easily be interfered by other information, so that the target information cannot be accurately extracted (Zoccatelli et al., 2010). Judgment test can be used as a method to monitor the information processing speed of brain aging, but there is still no objective evidence to explore the mechanism of brain aging from behavior. Therefore, this study introduced synchronous fNIRS technology to explore the functional interaction between cognitive related brain regions.

### 4.2. The influence of brain aging on the brain network of cognitive control tasks

This study found that the FC of homologous ROI in the elderly group was weaker than that in the young group in the forward and reverse judgment tests, which indicated that the FC of bilateral PFC and PMC decreased in the elderly group during cognitive tasks, suggesting that the function of brain network in cognitive and motor-related brain regions was weakened. Zhang et al. (2021) proposed that the increase of age was negatively related to the FC of the whole brain network, while the decrease of the FC of the brain network was related to cognitive behavior disorder, which supported the experimental results of this study. A large number of studies have shown that the FC values of age-related areas decrease in the default mode network, salience network and frontoparietal control network (Grady, 2012). Age-related functional brain network changes accelerate cognitive decline, and brain functional reorganization related to brain aging may be the key sign of cognitive aging (Zhao et al., 2020). Li et al. (2018) conducted fNIRS resting whole brain analysis on young people and old people. The results showed that the whole brain and local brain functions of old people decreased, and the results of small world analysis also showed a downward trend. However, young people showed rich hubs in prefrontal cortex, while the display hubs of old people shifted to sensory motor cortex. Therefore, brain aging not only affects local brain regions, but also affects the interaction of brain network functions among brain regions.

### 4.3. The influence of brain aging on pFC-related brain network

The FC value of heterologous ROI mainly reflects the FC strength of brain network between different ROI brain regions. The decrease of brain network connection strength between different brain regions leads to the decrease of cognitive control, which is the expression of the loss of overall integration function (Bishop et al., 2010). In this study, it was found that in the forward judgment test, except LPMC-LPFC, the heterologous ROI of PMC and PFC in the elderly group was significantly lower than that in the young group. However, in the reverse judgment test, only the heterogenous ROI of LPMC-RPFC, LPMC-LPFC, and RPFC-LPFC in the elderly group was significantly lower than that in the young group, which indicated that the interaction function of most PFC and PMC brain networks decreased in the forward judgment test, while that of both PFC and LPMC brain networks decreased in the reverse judgment test, suggesting that the interaction between PFC and PMC brain networks decreased in the simple cognitive task of the elderly, but there was a certain compensatory ability after the task difficulty was increased. Judgment task is a classic paradigm that is most often used to study top-down cognitive control processing, and it is mainly used to study selective attention and response inhibition function (Aguerre et al., 2021). Top-down cognitive control processing is accomplished by mobilizing, coordinating and directing subcortical and posterior cortical areas through the core carrier PFC (Aguerre et al., 2021). Sirawaj Itthipuripat et al. (2019) have found that people’s information processing is highly automated when people look at familiar stimuli, but PFC is a key brain area that plays a role in cognitive interference ability and reducing the trend of high automation. Functional magnetic resonance imaging (fMRI) shows that the functional changes of prefrontal lobe are related to normal brain aging (Cabeza et al., 2018). A large number of studies have shown that the aging of frontal lobe is related to the deterioration of executive function, indicating that frontal lobe is crucial to cognitive control (Fisher et al., 2014; Cipolotti et al., 2015; Rieck et al., 2021). Zheng et al. (2014) found that the right inferior frontal gyrus was related to the response inhibition task. In this study, it was found that only the heterologous brain network RPMC-RPFC in the elderly group in the reverse judgment test was significantly stronger than in the forward judgment test, and there was no significant difference in the FC of each brain network channel in the left ROI, showing a tendency of lateralization, suggesting that the enhancement of RPMC-RPFC brain network connection may be related to cognitive compensation. Yeung and Chan (2021) have shown that aging is related to the increased compensatory activity of frontal lobe in multiple task areas. The activation of PFC-related network on the right side may be related to visual processing, Brosnan et al. (2018) have found that the activation of PFC on the right side is related to the processing speed of visual information, which may be a sign of age-related decline. Therefore, the brain network interaction mode between PFC and other brain regions in the elderly has certain brain aging characteristics.

## 5. Limitations and prospects

This study explores the characteristics of brain network of brain aging from the cognitive perspective, and provides a direction for follow-up research. However, this study failed to cover more brain regions to explore the FC mechanism of brain network. In the future research, it is hoped that the research scope of brain regions can be further expanded, and the longitudinal research on the improvement mechanism of brain aging can be integrated to further explore the functional remodeling mechanism of brain aging, so as to promote the targeted assessment and rehabilitation of degenerative diseases of the elderly, and achieve early diagnosis, early treatment and early rehabilitation.

## 6. Summary

In this study, fNIRS synchronous judgment test was used to reveal the brain network mechanism of cognitive control of brain aging. It was found that the FC of brain network of the elderly in bilateral PFC and PMC was significantly lower than that of the young people, and there might be a tendency of lateralization, suggesting that brain aging is a kind of deterioration of whole brain function, which can reduce the speed of information processing and form a connection mode of brain network function different from that of the young people.

## Data availability statement

The original contributions presented in this study are included in the article/supplementary material, further inquiries can be directed to the corresponding authors.

## Ethics statement

The studies involving human participants were reviewed and approved by the Administrative Committee of the Fifth Affiliated Hospital of Guangzhou Medical University (KY01-2021-03-20) and registered by China Clinical Trial Center (ChiCTR2200058088). The patients/participants provided their written informed consent to participate in this study.

## Author contributions

JL, HO, and WL designed the study. JL, JH, and WL drafted the manuscript. JL, WL, YW, and ZT performed data analysis. WL, YW, JL, ZL, and LZ collected the data. JL and HO approved the final version of the manuscript. All authors contributed to the article and approved the submitted version.
